# Cryptogamous Origin of Fever, Cholera, &c.

**Published:** 1849-05

**Authors:** 


*Cryptogamous origin of Fever, Cholera, &c.*—The following are the conclusions at which Prof. J. K. Mitchell arrives in his work on the Cryptogamous origin of endemic and epidemic fevers, cholera, &c:—

"While I was impressed," (writes Dr. Mitchell,) "for the reason so ably stated by Holland with the greater probability of the organic theory, I prefer, for reasons stated by myself, the fungous, to the animalcular hypothesis.

"My preference is founded on the vast number, extraordinary variety, minuteness, diffusion and climatic peculiarities of the fungi.

"The spores of these plants are not only numerous, minute, and indefinitely diffused, but they are so like to animal cells, as to have the power of penetrating into, and germinating upon, the most interior tissues of the human body.

"Introduced into the body through the stomach, or by the skin or lungs, cryptogamous poisons were shown to produce diseases of a febrile character, intermittent, remittent and continued; which were most successfully treated by wine and bark.

"Many cutaneous diseases, such as *favus* and *mentagra*, are proved to be dependent upon cryptogamous vegetations; and even the disease of the mucous membrane, termed apthæ, arises from the presence of the minute fungi.

"As microscopic investigations become more minute, we discover protophytes in diseases, where, until our own time, their existence was not even suspected, as in the discharges of some kind of dysentery, and in the *sarcina* of pyrosis. We are therefore entitled to believe that discovery will be, on this subject, progressive.

"The detection of the origin of the muscardine of the silk worm, and a great many analogous diseases of insects, fishes and reptiles, and the demonstration of cryptogamism of these maladies, their contagious character in one species of animals, their transfer to many other species, nay even to vegetables themselves, all concur to render less improbable, the agency of fungi in the causation of diseases of a febrile character.

"A curious citation was subsequently made, of the fungiferous condition during epidemics and epizootics. These moulds, red, white, yellow, gray, or even black, stained garments, utensils and pavements, made the fogs fetid, and caused disagreeable odors and spots, even in the recesses of closets, and the interior of trunks and desks.

"These moulds existed, even when the hygrometric state did not give to the air any unusual moisture for their sustenation and propagation. Their germs seemed to have, as have epidemics, an inherent power of extension.

"The singular prevalence of malarious diseases in the autumn, is best explained by supposing them to be produced by the fungi, which grow most commonly at the same season. The season of greatest photophytic activity, is, in every country, the period of the greatest malarious disturbance. The sickly season is, in the rains in Africa, in the very dry season in Majorca and Sardinia, in the rainy season of the insular West Indies, and in the dry season of Demerara and Surinam. Even when the vegetation is peculiarly controlled, as in Egypt by the Nile, and the cryptogami are thus thrown into the season of winter and spring, that season becomes, contrary to rule, the pestilential part of the year.

"Marshes are a safe residence by day, whilst they are often highly dangerous by night. In the most deadly localities of our southern country, and of Africa, the sportsman may tread the mazes of a swamp safely by day, although at every step, he extricates vast quantities of the gases, which lie entangled in mud and vegetable mould. This point, so readily explained by reference to the acknowledged nocturnal growth and power of the fungi, is a complete stumbling-block to the miasmatists.

"The cryptogamous theory well explains the obstruction to the progress of malaria offered by a road, a wall, a screen of trees, a veil or gauze curtain.

"It also accounts for the nice localization of an ague, or yellow fever, or cholera, and the want of power in steady winds to convey malarious diseases into the heart of a city, from the adjacent country.

"It explains also well, the security afforded by artificially drying the air of malarious places, the exemption of cooks and smiths from the sweating sickness, the cause of the danger from mouldy sheets, and of the sternutation from old books and papers.

"On no other theory can we so well account, if account at all, for the phenomena of milzbrand and milk-sickness, the introduction of yellow fever into northern ports, and the wonderful irregularities of the progress of cholera.

"The cryptogamous theory will well explain the peculiar domestication of different diseases in different regions, which have a smilar climate; the plague of Egypt, the yellow fever of the Antilles, and the cholera of India. It accounts, too, for their occasional expansion into unaccustomed places, and their retreat back to their original haunts.

"Our hypothesis will also enable us to tell, why malarious sickness is disproportionate to the character of the seasons; why the dry Maremma abounds with fevers, while the wet shores of Brazil and Australia actually luxuriate in healthfulness. The prolonged incubative period, the frequent relapses of intermittents, and the latency of the malarious poisons for months can only be well explained by adopting the theory of a fungous causation.

"Finally, it explains the cause of the non-recurrence of very potent maladies, better than the chemical theory of Liebig; and shows why the earliest cases of an epidemic are commonly the most fatal."—*Med. Examiner.*

